# Association Between Hydrosalpinx and Detection of Gram‐Positive Anaerobic Cocci in the Endometrial Microbiota: A Single‐Center Retrospective Observational Study

**DOI:** 10.1002/rmb2.70109

**Published:** 2026-10-25

**Authors:** Ryota Okamoto, Noritoshi Enatsu, Hiroaki Shibahara, Shoji Kokeguchi, Masahide Shiotani

**Affiliations:** ^1^ Hanabusa Women's Clinic Kobe Hyogo Japan

**Keywords:** 16S rRNA gene sequencing, endometrial microbiota, gram‐positive anaerobic cocci, hydrosalpinx, infertility

## Abstract

**Purpose:**

To investigate whether hydrosalpinx is associated with alterations in the endometrial microbiota and detection of gram‐positive anaerobic cocci (GPAC)‐associated genera.

**Methods:**

This single‐center retrospective observational study included 236 patients who underwent endometrial microbiota testing by 16S ribosomal RNA gene sequencing: 90 with untreated hydrosalpinx diagnosed by hysterosalpingography and 146 without hydrosalpinx. GPAC positivity was defined as detection of at least one of *Anaerococcus*, *Finegoldia*, *Peptoniphilus*, *Peptostreptococcus*, or *Parvimonas*. Logistic regression was adjusted for age, antibiotic use within 3 months, and luteal‐phase sampling. False discovery rate adjustment was applied across four exploratory microbiota outcomes.

**Results:**

GPAC‐associated genera were detected in 23/90 patients (25.6%) with hydrosalpinx and 19/146 (13.0%) without hydrosalpinx. Hydrosalpinx was nominally associated with GPAC detection (adjusted odds ratio, 2.20; 95% confidence interval, 1.10–4.38; *p* = 0.025), but not after false discovery rate adjustment (*q* = 0.101). No clear associations were observed for the other microbiota outcomes. Similar estimates were obtained after additional adjustment for recurrent implantation failure and in post hoc sensitivity analyses using alternative GPAC definitions.

**Conclusion:**

Hydrosalpinx was associated with more frequent detection of GPAC‐associated genera in exploratory analyses. This finding should be considered hypothesis‐generating rather than confirmatory and requires independent prospective validation.

## 1 Introduction

Hydrosalpinx is characterized by fluid accumulation within a fallopian tube secondary to distal tubal obstruction and is associated with reduced implantation, clinical pregnancy, and live birth rates, as well as an increased risk of early pregnancy loss following in vitro fertilization (IVF) and embryo transfer [1]. Salpingectomy or proximal tubal occlusion before IVF may improve clinical pregnancy rates compared with no treatment [2]. Proposed mechanisms by which hydrosalpinx impairs pregnancy include mechanical interference caused by reflux of hydrosalpingeal fluid into the uterine cavity [3], inflammatory and immune alterations of the endometrial environment [4, 5], and impaired endometrial receptivity [6]. The reported increase in endometrial leukemia inhibitory factor expression during the implantation window after salpingectomy further suggests that hydrosalpinx may alter the endometrial environment [6].

Advances in molecular techniques, including 16S ribosomal RNA (rRNA) gene sequencing, have enabled investigation of the association between the endometrial microbiota and reproductive outcomes. A *Lactobacillus*‐dominated microbiota (LDM), commonly defined as a relative abundance of *Lactobacillus* of at least 90%, has been associated with favorable implantation and pregnancy outcomes [7, 8]. However, other studies have not demonstrated a clear association between *Lactobacillus* predominance and IVF outcomes [9, 10]. Furthermore, because the endometrium represents a low‐biomass environment, the effects of sampling procedures, analytical methods, and contamination require careful consideration [11]. Therefore, dichotomization based solely on the relative abundance of *Lactobacillus* may not adequately capture associations between specific non‐*Lactobacillus* bacterial groups and disorders of the female reproductive tract.

Gram‐positive anaerobic cocci (GPAC) comprise a heterogeneous group of bacteria that includes *Anaerococcus*, *Finegoldia*, *Peptoniphilus*, *Peptostreptococcus*, and *Parvimonas*. These organisms may be present as commensals on the skin and mucosal surfaces but can act as opportunistic pathogens under certain conditions [12]. Bacterial vaginosis (BV) is a polymicrobial dysbiosis characterized by an increase in diverse anaerobic bacteria, including *Gardnerella*, *Atopobium*, and *Prevotella* [13]. GPAC have also been associated with acute endometritis in women with pelvic inflammatory disease [14], and an increased relative abundance of several non‐*Lactobacillus* taxa, including *Anaerococcus*, has been reported in women with chronic endometritis [15]. However, the direct association between hydrosalpinx and the endometrial microbiota, including GPAC, remains insufficiently investigated.

A recent study of the female reproductive tract microbiota in women with hydrosalpinx identified a more dysbiotic vaginal microbiota, whereas bacterial signals obtained from excised fallopian tubes were limited [16]. In addition, a shotgun metagenomic sequencing study including 11 women with hydrosalpinx reported lower microbial content, assessed as the proportion of microbial‐derived reads among clean sequencing reads, and lower alpha diversity in uterine cavity samples from women with hydrosalpinx, although no clear between‐group difference in the relative abundance of *Lactobacillus* was observed [17]. These findings suggest that alterations in the intrauterine environment associated with hydrosalpinx may not be explained solely by a reduction in *Lactobacillus*.

In this study, we compared the endometrial microbiota according to the presence or absence of untreated hydrosalpinx diagnosed by hysterosalpingography. We evaluated the relative abundance of *Lactobacillus*, LDM, non‐*Lactobacillus*‐predominant microbiota (NLPM), selected representative BV‐associated genera, and detection of GPAC‐associated genera to explore microbiota signals associated with hydrosalpinx.

## 2 Materials and Methods

### 2.1 Study Design and Participants

This single‐center retrospective observational study was conducted at Hanabusa Women's Clinic. Patients who underwent endometrial microbiota testing using 16S rRNA gene sequencing performed by Varinos Inc. (Tokyo, Japan) between December 2022 and March 2026 were eligible.

When a patient had undergone endometrial microbiota testing more than once, only the first test was included to avoid within‐patient duplication. Patients whose bacterial signal was considered insufficient by the testing laboratory to evaluate microbiota composition and patients who had undergone bilateral salpingectomy before endometrial microbiota testing were excluded.

Eligible patients were classified into hydrosalpinx and no‐hydrosalpinx groups according to the presence of hydrosalpinx at the time of endometrial microbiota testing. No a priori sample size calculation was performed, and all patients who met the eligibility criteria during the study period were included.

### 2.2 Diagnosis of Hydrosalpinx

Hydrosalpinx was diagnosed based on hysterosalpingographic findings obtained before endometrial microbiota testing. Hysterosalpingography was performed using iotrolan contrast medium (Isovist Injection 300, 300 mg iodine/mL; Bayer Yakuhin Ltd., Osaka, Japan) diluted 1:1 with sterile 0.9% saline. Hydrosalpinx was defined as tubal dilatation consistent with hydrosalpinx accompanied by retention of contrast medium within the dilated segment on delayed imaging performed 20 min after contrast administration. Hydrosalpinx laterality was classified as unilateral or bilateral based on hysterosalpingographic findings.

Transvaginal ultrasonography was performed as part of routine clinical care; however, hysterosalpingography was the primary basis for group classification in this study. Patients with persistent hydrosalpinx at the time of endometrial microbiota testing were assigned to the hydrosalpinx group, whereas those without hydrosalpinx on hysterosalpingography were assigned to the no‐hydrosalpinx group.

None of the patients included in the hydrosalpinx group had undergone salpingectomy, proximal tubal occlusion, or any other intervention for hydrosalpinx before endometrial sample collection.

### 2.3 Endometrial Sample Collection and Microbiota Analysis

Endometrial samples were collected outside the menstrual period. After the vagina was cleansed with benzalkonium chloride, it was thoroughly rinsed with sterile saline, and cervical mucus around the ectocervix and external cervical os was removed. An endometrial suction curette (Pipet Curet; CooperSurgical Inc., Trumbull, CT, USA) was then introduced into the uterine cavity without contact with the vaginal wall, and an endometrial sample was collected. Samples were transferred to dedicated containers containing preservation solution and sent to Varinos Inc.

Bacterial DNA extraction, 16S rRNA gene sequencing, quality control, and contaminant removal were performed at Varinos Inc. according to the method reported by Hanaoka et al. [18]. The same analytical protocol was used throughout the study period, from sample processing through taxonomic classification and contaminant removal.

The V1–V2 region of the 16S rRNA gene was amplified from extracted DNA, followed by 251‐bp paired‐end sequencing using the Illumina MiSeq platform. After quality control, operational taxonomic units were generated at 99.5% sequence similarity using QIIME version 1.9.1. Taxonomic classification was performed using BLAST against a database constructed from SILVA 138 and STIRRUPS. Sequences with less than 99% similarity to the reference database or an alignment length covering less than 95% of the full sequence were excluded.

Positive controls with known bacterial composition and negative controls consisting of sterile water were included in the analytical workflow. Bacterial taxa considered to originate from reagents or the laboratory environment were removed [18]. The detected bacterial taxa and their relative abundances were calculated for each sample.

### 2.4 Definitions of Endometrial Microbiota Outcomes

The relative abundance of *Lactobacillus* in each sample was calculated as the sum of the relative abundances of all species classified within the genus *Lactobacillus*. Based on previous studies, LDM was defined as a relative abundance of *Lactobacillus* of at least 90%, whereas non‐*Lactobacillus*‐dominated microbiota (NLDM) was defined as a relative abundance of less than 90% [7].

The predominant bacterial species was defined as the species with the highest relative abundance in each sample. A sample was classified as NLPM when the predominant species did not belong to the genus *Lactobacillus*.

For the selected representative BV‐associated genera, positivity was defined as detection of at least one of *Gardnerella*, *Atopobium*, or *Prevotella* in the laboratory report [13]. Each genus was also evaluated separately.

### 2.5 Definitions of GPAC‐Associated Genera and GPAC Positivity

In this study, the five genera *Anaerococcus*, *Finegoldia*, *Peptoniphilus*, *Peptostreptococcus*, and *Parvimonas*, which have been described as major GPAC genera isolated from human infections, were selected as the GPAC‐associated genera evaluated in the present analysis [12]. GPAC positivity was defined as detection of at least one of these five genera in the laboratory report. No additional relative‐abundance threshold was applied to this definition.

### 2.6 Post Hoc Sensitivity Analyses of GPAC

To assess whether the direction of the association was maintained after excluding very‐low‐abundance bacterial signals, the total GPAC relative abundance was calculated as the sum of the relative abundances of the five GPAC‐associated genera. Post hoc sensitivity analyses were performed using total GPAC relative‐abundance thresholds of at least 0.5% and at least 1.0%. These thresholds were selected to evaluate analytical robustness and were not based on established biological or clinical criteria. To reduce the potential influence of low‐level detection of a single genus, a further post hoc sensitivity analysis defined positivity as detection of at least two of the five GPAC‐associated genera.

A leave‐one‐genus‐out sensitivity analysis was also performed to assess whether the association between the composite GPAC indicator and hydrosalpinx was disproportionately dependent on any single genus. In each analysis, one of the five genera was omitted and the composite indicator was redefined using the remaining four genera.

### 2.7 Clinical Characteristics

Patient characteristics and clinical information at the time of testing were retrospectively obtained from medical records. Evaluated variables included age at testing, primary or secondary infertility, history of miscarriage, recurrent miscarriage, previous live birth, recurrent implantation failure (RIF), endometriosis, documented chlamydial test positivity, antibiotic use within 3 months before testing, timing of sample collection, previous hysteroscopy, and hysteroscopic findings suggestive of chronic endometritis.

Recurrent miscarriage was defined as two or more consecutive miscarriages before 22 weeks of gestation.

RIF was defined as failure to achieve a clinical pregnancy despite transfer of at least two good‐quality blastocysts, including embryo transfers performed at other institutions. A good‐quality blastocyst was defined as a blastocyst with an expansion grade of at least 3 and inner cell mass and trophectoderm grades of AA, AB, BA, or BB according to the Gardner classification [19]. Patients for whom the morphology of previously transferred embryos or treatment details could not be confirmed from the medical record were treated as having missing data for RIF.

Antibiotic exposure was defined as documented antibiotic use within 3 months before endometrial microbiota testing. Patients for whom antibiotic use could not be confirmed from the medical record were treated as having missing data for this variable. The timing of sample collection was classified as luteal phase or other based on the medical record.

Chlamydia‐related test results obtained before endometrial microbiota testing were retrospectively collected from the medical records. Serum *Chlamydia trachomatis* IgG and IgA antibodies were measured by enzyme immunoassay, and testing for *C. trachomatis* DNA was performed using real‐time polymerase chain reaction. All tests were performed at SRL Inc. (Tokyo, Japan). Documented chlamydial test positivity was defined as a positive IgG antibody, IgA antibody, or *C. trachomatis* DNA test result obtained before endometrial microbiota testing. Patients with at least one evaluable test and no positive result were classified as having no documented positivity, whereas patients without an evaluable result or with only indeterminate results were treated as having missing data.

Hysteroscopic findings suggestive of chronic endometritis were evaluated among patients who had undergone hysteroscopy before endometrial microbiota testing, using findings from the most recent hysteroscopic examination before testing. Among the hysteroscopic findings available for evaluation in this study, the presence of at least one of the following was classified as suggestive of chronic endometritis: micropolyps, focal or diffuse endometrial hyperemia, or stromal edema [20]. Because histological assessment using CD138 immunostaining was not performed in all patients, these findings were distinguished from a definitive diagnosis of chronic endometritis. Patients who had not undergone hysteroscopy before endometrial microbiota testing were treated as having missing data for this variable.

### 2.8 Statistical Analysis

Continuous variables are presented as mean ± standard deviation or median [interquartile range], depending on their distributions. Age was compared between groups using Welch's *t*‐test. The relative abundance of *Lactobacillus* and total GPAC relative abundance were compared using the Mann–Whitney *U* test.

Categorical variables are presented as number and percentage. Fisher's exact test was used when any expected cell count was less than 5; otherwise, Pearson's chi‐square test without Yates' continuity correction was used. For variables with missing data, between‐group comparisons were based on patients for whom the relevant variable was available.

To evaluate associations between hydrosalpinx and endometrial microbiota outcomes, binary logistic regression analyses were performed with the presence of hydrosalpinx as the explanatory variable and NLDM, NLPM, positivity for any selected representative BV‐associated genus, and GPAC positivity as separate dependent variables. Model 1 was adjusted for age at testing, antibiotic use within 3 months before testing, and luteal‐phase sampling. RIF could be positioned on the causal pathway as a consequence of the adverse reproductive effects of hydrosalpinx, but it could also represent a confounding difference in patient background. Therefore, Model 2 additionally included RIF. Adjusted odds ratios (aORs) and 95% confidence intervals (CIs) were calculated for each model. To assess potential confounding by previous chlamydial infection or exposure, an additional sensitivity analysis was performed by adding documented chlamydial test positivity to Model 2. To allow direct comparison of the estimates, Model 2 was refitted using the same complete‐case population as that used for the model additionally adjusted for documented chlamydial test positivity.

No single primary microbiota outcome was prespecified. NLDM, NLPM, positivity for any selected representative BV‐associated genus, and GPAC positivity were evaluated as four parallel exploratory outcomes. For these four outcomes, the false discovery rate (FDR) was controlled separately within Model 1 and Model 2 using the Benjamini–Hochberg procedure, and adjusted *q* values were calculated [21].

Individual analyses of the five GPAC‐associated genera were treated as a separate exploratory family of tests. Because the number of detection events for each genus was limited, Firth bias‐reduced logistic regression was performed with adjustment for age at testing, antibiotic use within 3 months before testing, and luteal‐phase sampling [22]. *p* values obtained from the five genus‐level analyses were adjusted for multiple testing using the Benjamini–Hochberg procedure.

Analyses based on total GPAC relative‐abundance thresholds of at least 0.5% and at least 1.0%, detection of at least two GPAC‐associated genera, and leave‐one‐genus‐out definitions were performed as post hoc robustness analyses. These analyses used the same covariates as Model 1.

For the basic definition of GPAC positivity, total GPAC relative abundance of at least 0.5%, total GPAC relative abundance of at least 1.0%, and detection of at least two GPAC‐associated genera, conventional logistic regression and Firth bias‐reduced logistic regression were both performed. Conventional logistic regression was used for the leave‐one‐genus‐out analyses.

In these sensitivity analyses, consistency of the effect estimates and 95% CIs was emphasized. *p* values from the post hoc analyses and the Firth sensitivity analyses were considered descriptive and were not subjected to additional multiplicity adjustment.

Missing values were not imputed. Complete‐case analyses were performed using patients with all variables required for each model. All tests were two‐sided. A *p* value of less than 0.05 was considered nominally statistically significant, whereas an FDR‐adjusted *q* value of less than 0.05 was considered statistically significant after multiplicity adjustment. Statistical analyses were performed using R version 4.5.0 (R Foundation for Statistical Computing, Vienna, Austria).

### 2.9 Ethical Considerations

This study was approved by the Ethics Committee of Hanabusa Women's Clinic (Approval No. 2026‐08) and was conducted in accordance with the Declaration of Helsinki.

Comprehensive written informed consent for the potential secondary use of de‐identified clinical and test data for research and academic presentation was obtained from all patients. Because this retrospective observational study used existing clinical information and test results, the requirement for additional study‐specific informed consent was waived by the ethics committee.

## 3 Results

### 3.1 Study Population

During the study period, 281 endometrial microbiota tests were performed. When multiple tests had been performed in the same patient, only the first test was retained, resulting in 247 patients undergoing eligibility assessment. Six patients whose bacterial signals were considered insufficient by the testing laboratory to evaluate microbiota composition and five patients who had undergone bilateral salpingectomy before endometrial microbiota testing were excluded. A total of 236 patients were included in the final analysis: 90 in the hydrosalpinx group and 146 in the no‐hydrosalpinx group (Figure 1). Among the five patients excluded because they had undergone bilateral salpingectomy before endometrial microbiota testing, none had detectable GPAC‐associated genera. This finding was descriptive and was not included in the comparative analyses.

**FIGURE 1:**
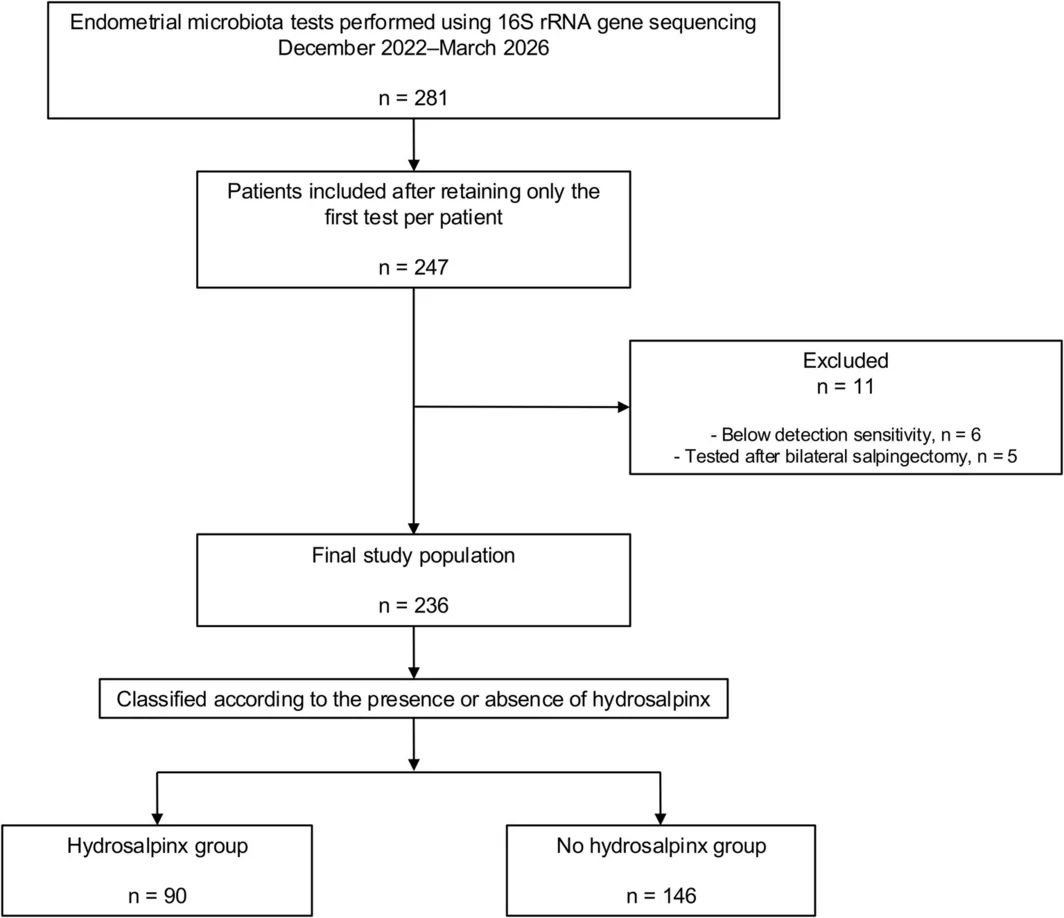
Flow diagram of patient selection. Among 281 endometrial microbiota tests performed using 16S ribosomal RNA gene sequencing between December 2022 and March 2026, only the first test per patient was retained, resulting in 247 patients undergoing eligibility assessment. Six patients whose bacterial signals were insufficient to evaluate microbiota composition and five patients who had undergone bilateral salpingectomy before endometrial microbiota testing were excluded. A total of 236 patients were included in the final analysis: 90 with hydrosalpinx and 146 without hydrosalpinx.

### 3.2 Patient Characteristics

The mean age was 36.0 ± 4.4 years in the hydrosalpinx group and 36.6 ± 4.7 years in the no‐hydrosalpinx group, with no significant between‐group difference (*p* = 0.316). Among the 90 patients with hydrosalpinx, 57 (63.3%) had unilateral and 33 (36.7%) had bilateral hydrosalpinx. RIF was observed in 67 of 89 evaluable patients (75.3%) in the hydrosalpinx group and 72 of 141 evaluable patients (51.1%) in the no‐hydrosalpinx group and was more frequent in the hydrosalpinx group (*p* < 0.001) (Table 1). No significant between‐group differences were observed in the distribution of primary and secondary infertility, history of miscarriage, recurrent miscarriage, previous live birth, endometriosis, antibiotic use within 3 months before testing, or the proportion of samples collected during the luteal phase.

**TABLE 1:** Clinical characteristics according to hydrosalpinx status.

Characteristic	Hydrosalpinx (*n* = 90)	No hydrosalpinx (*n* = 146)	*p*
Age, years	36.0 ± 4.4	36.6 ± 4.7	0.316
Primary infertility	26 (28.9)	43 (29.5)	0.926
History of miscarriage	39 (43.3)	57 (39.0)	0.514
Recurrent miscarriage	17 (18.9)	28 (19.2)	0.956
Previous live birth	42 (46.7)	75 (51.4)	0.483
Recurrent implantation failure	67/89 (75.3)	72/141 (51.1)	< 0.001
Endometriosis	7 (7.8)	13 (8.9)	0.763
Documented chlamydial test positivity	15/87 (17.2)	22/141 (15.6)	0.854
Antibiotic use in the preceding 3 months	22/88 (25.0)	41/141 (29.1)	0.501
Sampling during the luteal phase	55 (61.1)	79 (54.1)	0.292
Hysteroscopy performed before sampling	89 (98.9)	137 (93.8)	0.094
Hysteroscopic findings suggestive of chronic endometritis	52/89 (58.4)	82/137 (59.9)	0.831

*Note:* Data are presented as mean ± standard deviation, *n* (%), or *n/N* (%), where *N* is the number with available data. Recurrent miscarriage was defined as two or more consecutive miscarriages before 22 weeks of gestation; recurrent implantation failure as no clinical pregnancy after ≥ 2 good‐quality blastocyst transfers. Documented chlamydial test positivity was defined as positivity for serum *C. trachomatis* IgG or IgA antibodies or *C. trachomatis* DNA before endometrial microbiota testing. *p* values were calculated using Welch's *t*‐test, Pearson's chi‐square test, or Fisher's exact test, as appropriate.

Hysteroscopy had been performed before endometrial microbiota testing in 89 of 90 patients (98.9%) in the hydrosalpinx group and 137 of 146 patients (93.8%) in the no‐hydrosalpinx group, with no significant between‐group difference (*p* = 0.094). Among patients who underwent hysteroscopy, findings suggestive of chronic endometritis were observed in 52 of 89 patients (58.4%) and 82 of 137 patients (59.9%), respectively, with no significant between‐group difference (*p* = 0.831). Chlamydial test results were evaluable in 228 patients. Documented chlamydial test positivity was identified in 15 of 87 evaluable patients (17.2%) in the hydrosalpinx group and 22 of 141 (15.6%) in the no‐hydrosalpinx group, with no significant difference (*p* = 0.854) (Table 1). Among the 37 patients with documented positivity, 33 were IgG antibody‐positive and 19 were IgA antibody‐positive; no positive *C. trachomatis* DNA result was identified.

### 3.3 Comparison of the Endometrial Microbiota According to Hydrosalpinx Status

The median relative abundance of *Lactobacillus* was 91.3% [interquartile range, 26.0%–99.7%] in the hydrosalpinx group and 94.2% [6.2%–99.6%] in the no‐hydrosalpinx group, with no significant between‐group difference (*p* = 0.716). NLDM was observed in 45 of 90 patients (50.0%) in the hydrosalpinx group and 70 of 146 patients (47.9%) in the no‐hydrosalpinx group, with no significant between‐group difference (*p* = 0.759). NLPM was observed in 11 of 90 patients (12.2%) and 22 of 146 patients (15.1%), respectively, with no significant between‐group difference (*p* = 0.540) (Table 2). Positivity for at least one of the selected representative BV‐associated genera, *Gardnerella*, *Atopobium*, or *Prevotella*, was observed in 44 of 90 patients (48.9%) in the hydrosalpinx group and 70 of 146 patients (47.9%) in the no‐hydrosalpinx group, with no significant between‐group difference (*p* = 0.888). The detection frequencies of *Gardnerella*, *Atopobium*, and *Prevotella* considered separately also did not differ significantly between groups.

**TABLE 2:** Endometrial microbiota characteristics according to hydrosalpinx status.

Microbiota characteristic	Hydrosalpinx (*n* = 90)	No hydrosalpinx (*n* = 146)	*p*
*Lactobacillus* relative abundance (%)	91.3 [26.0–99.7]	94.2 [6.2–99.6]	0.716
Non‐*Lactobacillus*‐dominated microbiota	45 (50.0)	70 (47.9)	0.759
Non‐*Lactobacillus*‐predominant microbiota	11 (12.2)	22 (15.1)	0.540
Detection of any representative BV‐associated genus	44 (48.9)	70 (47.9)	0.888
*Gardnerella*	25 (27.8)	40 (27.4)	0.949
*Atopobium*	8 (8.9)	25 (17.1)	0.076
*Prevotella*	25 (27.8)	32 (21.9)	0.307
Detection of any GPAC genus	23 (25.6)	19 (13.0)	0.014
*Anaerococcus*	10 (11.1)	3 (2.1)	0.006
*Finegoldia*	16 (17.8)	12 (8.2)	0.027
*Peptoniphilus*	15 (16.7)	8 (5.5)	0.005
*Peptostreptococcus*	5 (5.6)	2 (1.4)	0.109
*Parvimonas*	0 (0.0)	2 (1.4)	0.526

*Note:* Data are presented as median [interquartile range] or *n* (%). Non‐*Lactobacillus*‐dominated microbiota was defined as a *Lactobacillus* relative abundance of < 90%. Non‐*Lactobacillus*‐predominant microbiota was defined as microbiota in which the predominant species did not belong to the genus *Lactobacillus*. Representative bacterial vaginosis‐associated genera included *Gardnerella*, *Atopobium*, and *Prevotella*. GPAC‐associated genera were operationally defined as *Anaerococcus*, *Finegoldia*, *Peptoniphilus*, *Peptostreptococcus*, and *Parvimonas*. *p* values were calculated using the Mann–Whitney *U* test, Pearson's chi‐square test, or Fisher's exact test, as appropriate. The genus‐level comparisons presented in this table were unadjusted and exploratory; adjusted Firth logistic regression estimates and false discovery rate‐adjusted *q* values are presented in Table S3.

Abbreviations: BV, bacterial vaginosis; GPAC, Gram‐positive anaerobic cocci; IQR, interquartile range.

In contrast, at least one GPAC‐associated genus was detected in 23 of 90 patients (25.6%) in the hydrosalpinx group and 19 of 146 patients (13.0%) in the no‐hydrosalpinx group. Within the hydrosalpinx group, GPAC‐associated genera were detected in 14 of 57 patients (24.6%) with unilateral hydrosalpinx and 9 of 33 patients (27.3%) with bilateral hydrosalpinx. The unadjusted detection frequency was higher in the hydrosalpinx group (*p* = 0.014).

### 3.4 Multivariable Analysis

The complete‐case analysis for Model 1, adjusted for age at testing, antibiotic use within 3 months before testing, and luteal‐phase sampling, included 229 patients. Hydrosalpinx was not associated with NLDM (aOR, 1.02; 95% CI: 0.60–1.75; *p* = 0.935; *q* = 0.935), NLPM (aOR, 0.79; 95% CI: 0.36–1.74; *p* = 0.562; *q* = 0.935), or positivity for any selected representative BV‐associated genus (aOR, 0.97; 95% CI: 0.56–1.66; *p* = 0.901; *q* = 0.935). In contrast, hydrosalpinx was nominally associated with GPAC detection (aOR, 2.20; 95% CI: 1.10–4.38; *p* = 0.025). However, this association did not meet the threshold for statistical significance after FDR adjustment across the four microbiota outcomes (*q* = 0.101) (Figure 2).

**FIGURE 2:**
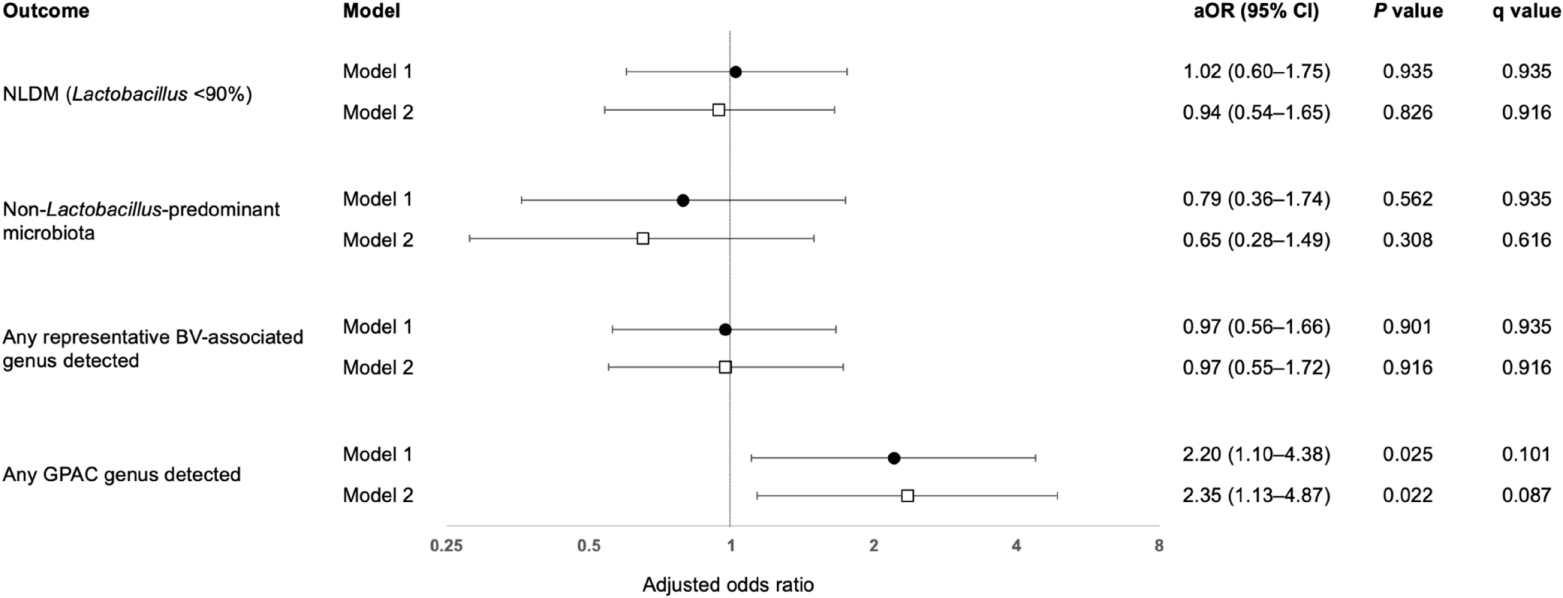
Adjusted associations between hydrosalpinx and endometrial microbiota outcomes. Filled circles indicate estimates from Model 1, and open squares indicate estimates from Model 2. Horizontal lines represent 95% confidence intervals. Model 1 was adjusted for age, antibiotic use within 3 months before testing, and luteal‐phase sampling and included 229 complete cases. Model 2 was additionally adjusted for recurrent implantation failure and included 223 complete cases. The Benjamini–Hochberg procedure was applied separately within each model across the four microbiota outcomes, and the resulting *q* values are presented. An adjusted odds ratio greater than 1 indicates higher odds of the corresponding outcome in patients with hydrosalpinx. AOR, adjusted odds ratio; BV, bacterial vaginosis; CI, confidence interval; GPAC, Gram‐positive anaerobic cocci; NLDM, non‐*Lactobacillus*‐dominated microbiota; NLPM, non‐*Lactobacillus*‐predominant microbiota.

The complete‐case analysis for Model 2, which additionally adjusted for RIF, included 223 patients. Hydrosalpinx was not associated with NLDM (aOR, 0.94; 95% CI: 0.54–1.65; *p* = 0.826; *q* = 0.916), NLPM (aOR, 0.65; 95% CI: 0.28–1.49; *p* = 0.308; *q* = 0.616), or positivity for any selected representative BV‐associated genus (aOR, 0.97; 95% CI: 0.55–1.72; *p* = 0.916; *q* = 0.916). For GPAC detection, a similar effect estimate was obtained after additional adjustment for RIF (aOR, 2.35; 95% CI: 1.13–4.87; *p* = 0.022), although the association did not meet the threshold for statistical significance after FDR adjustment (*q* = 0.087) (Figure 2).

In an additional sensitivity analysis of the same 216 complete cases, the aOR for the association between hydrosalpinx and GPAC detection was 2.67 (95% CI: 1.26–5.65; *p* = 0.010) in the refitted Model 2 and 2.68 (95% CI: 1.26–5.67; *p* = 0.010) after additional adjustment for documented chlamydial test positivity.

### 3.5 Post Hoc Sensitivity Analyses Using Alternative GPAC Definitions

A total GPAC relative abundance of at least 0.5% was observed in 12 of 90 patients (13.3%) in the hydrosalpinx group and 8 of 146 patients (5.5%) in the no‐hydrosalpinx group. In the analysis adjusted for the same covariates as Model 1, hydrosalpinx was associated with higher odds of a total GPAC relative abundance of at least 0.5% (aOR, 2.75; 95% CI: 1.07–7.07; *p* = 0.036). A total GPAC relative abundance of at least 1.0% was observed in 9 of 90 patients (10.0%) and 5 of 146 patients (3.4%), respectively. The effect estimate remained in the same direction when the 1.0% threshold was applied (aOR, 3.41; 95% CI: 1.09–10.70; *p* = 0.036). Detection of at least two GPAC‐associated genera was observed in 12 of 90 patients (13.3%) in the hydrosalpinx group and 6 of 146 patients (4.1%) in the no‐hydrosalpinx group. In the adjusted analysis, hydrosalpinx was associated with higher odds of detection of at least two GPAC‐associated genera (aOR, 3.51; 95% CI: 1.26–9.78; *p* = 0.016) (Figure S1).

Firth bias‐reduced logistic regression yielded effect estimates similar to those obtained using conventional logistic regression. For the basic definition of GPAC positivity, the Firth model yielded an aOR of 2.15 (95% CI: 1.09–4.25; *p* = 0.027). The direction of the effect estimates was also consistent in analyses using the 0.5% threshold, the 1.0% threshold, and detection of at least two GPAC‐associated genera (Table S1).

### 3.6 Leave‐One‐Genus‐Out Sensitivity Analysis

The composite GPAC indicator was redefined by omitting each of the five genera in turn. When *Finegoldia* was omitted, 19 patients (21.1%) in the hydrosalpinx group and 12 patients (8.2%) in the no‐hydrosalpinx group were positive, and the adjusted association was maintained (aOR, 2.68; 95% CI: 1.22–5.92; *p* = 0.014). The association remained in the same direction when *Peptoniphilus* was omitted (aOR, 2.60; 95% CI: 1.24–5.46; *p* = 0.012), when *Peptostreptococcus* was omitted (aOR, 2.35; 95% CI: 1.17–4.74; *p* = 0.017), and when *Parvimonas* was omitted (aOR, 2.36; 95% CI: 1.17–4.75; *p* = 0.016). In contrast, when *Anaerococcus* was omitted, 19 patients (21.1%) in the hydrosalpinx group and 19 patients (13.0%) in the no‐hydrosalpinx group were positive. The effect estimate was attenuated and the 95% CI crossed 1 (aOR, 1.69; 95% CI: 0.83–3.45; *p* = 0.150) (Table S2).

### 3.7 Total GPAC Relative Abundance

In the overall study population, the median total GPAC relative abundance was 0% [0%–0.075%] in the hydrosalpinx group and 0% [0%–0%] in the no‐hydrosalpinx group, with a significant between‐group difference (*p* = 0.012). In a descriptive analysis restricted to GPAC‐positive patients, the median total GPAC relative abundance was 0.6% [0.25%–3.1%] in the hydrosalpinx group and 0.3% [0.1%–0.9%] in the no‐hydrosalpinx group, with no significant between‐group difference (*p* = 0.429).

### 3.8 Exploratory Genus‐Level Analyses of GPAC‐Associated Genera

*Anaerococcus* was detected in 10 of 90 patients (11.1%) in the hydrosalpinx group and 3 of 146 patients (2.1%) in the no‐hydrosalpinx group. *Finegoldia* was detected in 16 patients (17.8%) and 12 patients (8.2%), respectively; *Peptoniphilus* in 15 patients (16.7%) and 8 patients (5.5%); *Peptostreptococcus* in 5 patients (5.6%) and 2 patients (1.4%); and *Parvimonas* in 0 patients (0%) and 2 patients (1.4%).

In Firth logistic regression analyses adjusted for age at testing, antibiotic use within 3 months before testing, and luteal‐phase sampling, exploratory associations with hydrosalpinx were observed for *Anaerococcus* (aOR, 5.54; 95% CI: 1.66–18.43; *p* = 0.005; *q* = 0.026), *Finegoldia* (aOR, 2.53; 95% CI: 1.14–5.61; *p* = 0.023; *q* = 0.038), and *Peptoniphilus* (aOR, 2.91; 95% CI: 1.21–7.04; *p* = 0.018; *q* = 0.038). No clear associations were observed for *Peptostreptococcus* (aOR, 3.58; 95% CI: 0.87–14.74; *p* = 0.078; *q* = 0.097) or *Parvimonas* (aOR, 0.33; 95% CI: 0.03–4.15; *p* = 0.390; *q* = 0.390) (Table S3).

## 4 Discussion

In this study, GPAC‐associated genera were detected more frequently in the endometrial microbiota of patients with hydrosalpinx. In a model adjusted for age, recent antibiotic use, and timing of sample collection, hydrosalpinx was nominally associated with approximately twofold higher odds of GPAC detection. A similar effect estimate was obtained after additional adjustment for RIF. However, the association did not meet the 5% significance threshold after FDR adjustment across the four microbiota outcomes. The GPAC finding should therefore be interpreted as an exploratory signal requiring validation in an independent population rather than as a confirmatory association.

The direction of the effect estimates remained consistent when GPAC positivity was defined using total relative‐abundance thresholds of at least 0.5% or at least 1.0%, detection of at least two GPAC‐associated genera, and Firth bias‐reduced logistic regression. These findings suggest that the observed association may not have been attributable exclusively to detection at the lowest relative abundances. Nevertheless, these were post hoc analyses performed using the same dataset and do not constitute independent validation. Furthermore, because no additional multiplicity adjustment was applied, the individual *p* values should be interpreted descriptively.

In the leave‐one‐genus‐out analyses, the direction of the association was maintained after omission of *Finegoldia*, *Peptoniphilus*, *Peptostreptococcus*, or *Parvimonas*, whereas omission of *Anaerococcus* attenuated the effect estimate to 1.69 and the 95% CI crossed 1. In the genus‐level Firth regression analyses, *Anaerococcus*, *Finegoldia*, and *Peptoniphilus* remained associated with hydrosalpinx after FDR adjustment within the family of five genus‐level tests. These results suggest that the signal underlying the composite GPAC indicator was not distributed equally across the five genera and is consistent with a possible contribution of *Anaerococcus* to the observed difference in the composite outcome. However, the attenuation after omission of *Anaerococcus* may also have reflected the reduction in the number of positive events and the resulting loss of precision. Because the genus‐level analyses were exploratory, with small numbers of detection events and wide confidence intervals, no causal or pathogenic role can be assigned to any specific genus.

Although total GPAC relative abundance differed between groups in the overall study population, no clear difference was observed in the descriptive analysis restricted to GPAC‐positive patients. The observed between‐group difference may therefore have primarily reflected a difference in the proportion of patients in whom GPAC‐associated genera were detected rather than an increase in relative abundance among GPAC‐positive patients. Moreover, relative abundance derived from 16S rRNA gene sequencing does not represent absolute bacterial quantity, and an increase in relative abundance does not necessarily indicate an increase in absolute bacterial burden [23]. The present results should therefore not be interpreted as evidence of GPAC proliferation or an increase in absolute GPAC burden.

Notably, none of the five patients tested after bilateral salpingectomy had detectable GPAC‐associated genera. This descriptive observation is directionally consistent with the association identified in the main analysis and is therefore potentially hypothesis‐generating. However, the number of patients was extremely small, and paired preoperative endometrial microbiota samples were unavailable. No inference can therefore be made that salpingectomy eliminated GPAC‐associated genera or altered the endometrial microbiota. Longitudinal studies using paired samples collected before and after intervention for hydrosalpinx are required.

No clear association with hydrosalpinx was observed for the relative abundance of *Lactobacillus*, NLDM, or NLPM. Although the 90% threshold facilitates comparison with previous studies [7, 8], it has not been established as a universal clinical threshold distinguishing a normal from an abnormal endometrial microbiota [9, 10]. The absence of a difference in *Lactobacillus*‐related outcomes does not demonstrate that hydrosalpinx has no effect on the endometrial microbiota. Rather, broad measures of *Lactobacillus* predominance alone may not capture specific anaerobic bacterial signals associated with hydrosalpinx.

GPAC may be present on the mucosa of the female genitourinary tract and can act as opportunistic pathogens under certain conditions [12]. GPAC have been associated with acute endometritis in women with pelvic inflammatory disease [14], and increased relative abundance of several non‐*Lactobacillus* taxa, including *Anaerococcus*, has been reported in women with chronic endometritis [15]. These observations provide biological plausibility for the association observed in the present study. Nevertheless, detection of GPAC‐associated genera by 16S rRNA gene sequencing does not establish bacterial viability or activity, pathogenicity, or active infection, and GPAC positivity alone should not be considered an indication for antimicrobial treatment.

Previous studies examining the association between hydrosalpinx and the female reproductive tract microbiota remain limited. Yagel et al. reported vaginal dysbiosis in women with hydrosalpinx, whereas Li et al. reported lower proportions of microbial‐derived reads and lower alpha diversity in uterine cavity samples; neither study specifically focused on GPAC [16, 17]. The present study complements these findings by identifying a difference in the detection frequency of GPAC‐associated genera, distinct from broad *Lactobacillus*‐related outcomes, in a larger population of patients with hydrosalpinx.

Several mechanisms may explain the association between hydrosalpinx and GPAC detection. Repeated reflux of hydrosalpingeal fluid into the uterine cavity may alter the local endometrial environment [3], while hydrosalpinx has been associated with inflammatory and immune alterations in the endometrium [4, 5]. These alterations may provide a biological context for the observed difference in GPAC detection, although this mechanism was not directly assessed in the present study. Alternatively, hydrosalpinx and GPAC detection may both reflect a shared antecedent inflammatory or infectious condition. Additional adjustment for documented chlamydial test positivity did not materially alter the effect estimate, suggesting that the observed association was not substantially accounted for by documented previous chlamydial infection or exposure. Because this was a cross‐sectional retrospective analysis, it was not possible to determine whether hydrosalpinx promoted GPAC detection, whether previous exposure to GPAC or other bacteria contributed to the development of hydrosalpinx, or whether both findings reflected a shared underlying condition.

The frequency of hysteroscopic findings suggestive of chronic endometritis did not differ according to hydrosalpinx status. However, hysteroscopic findings alone do not establish a definitive diagnosis of chronic endometritis [20], and histological assessment including CD138 immunostaining was not performed in all patients. The present findings therefore do not exclude an association between hydrosalpinx or GPAC detection and histologically confirmed chronic endometritis.

The clinical relevance of this study lies in the possibility that alterations in the intrauterine environment associated with hydrosalpinx may manifest as an increased detection frequency of specific anaerobic bacterial groups rather than as a simple reduction in *Lactobacillus* predominance. However, reproductive outcomes such as implantation, pregnancy, live birth, and miscarriage were not evaluated. It therefore remains unknown whether GPAC detection mediates the adverse IVF outcomes associated with hydrosalpinx. The present findings also do not support the use of antibiotics or probiotics solely on the basis of GPAC positivity.

Strengths of this study include the relatively large number of patients with hydrosalpinx, consistent diagnosis based on hysterosalpingography, inclusion of hydrosalpinges that had not been treated before sample collection, use of the same microbiota analysis protocol throughout the study period, adjustment for relevant clinical variables, evaluation of multiplicity using FDR correction, and assessment of the robustness of the GPAC finding through multiple post hoc sensitivity analyses.

This study also has several limitations. First, it was a single‐center retrospective study restricted to patients who underwent endometrial microbiota testing. Selection bias may therefore have been present, and the generalizability of the findings to the broader infertile population is limited. The no‐hydrosalpinx group was not a healthy fertile control group and consisted of patients with other causes of infertility.

Second, hydrosalpinx was classified based on hysterosalpingography, but size, ultrasonographic visibility, etiology, and the interval between hysterosalpingography and sample collection were not evaluated in detail. Tubal histopathological findings were not systematically available and therefore could not be evaluated. Although documented chlamydial test positivity was assessed, antibody positivity alone could not establish the timing or activity of infection, and no positive *C. trachomatis* DNA result was identified in this study. Other causes of tubal damage and previous pelvic infections unrelated to chlamydia were not systematically assessed. Residual and unmeasured confounding therefore cannot be excluded.

Third, because the endometrium is a low‐biomass environment, the effects of transcervical sampling and reagent‐derived contamination cannot be completely excluded [11, 24]. Although vaginal cleansing, avoidance of contact with the vaginal wall, positive and negative controls, and removal of presumed contaminants were incorporated, these procedures cannot establish that every low‐abundance signal originated in vivo.

Fourth, 16S rRNA gene sequencing provides compositional information based on relative abundance and does not directly determine absolute bacterial quantity, viability, or metabolic activity [11, 23]. Furthermore, bacterial detection alone does not establish pathogenicity or the presence of a host inflammatory response. The basic definition of GPAC positivity was based on detection of any one of the five genera in the laboratory report and was not a clinically validated quantitative threshold. The 0.5% and 1.0% thresholds were also selected post hoc as analytical criteria.

Fifth, antibiotic exposure, RIF, and other clinical variables derived from medical records may have been affected by missing data or measurement error, and residual and unmeasured confounding cannot be excluded. Because RIF could represent either a confounder or a variable on the causal pathway, two adjusted models were presented; however, the causal structure cannot be established from the present data.

Sixth, no single primary microbiota outcome was prespecified, and multiple microbiota outcomes were evaluated exploratorily. The association with the composite GPAC indicator did not meet the significance threshold after FDR adjustment. Although some genus‐level *q* values were below 0.05, these findings were based on limited numbers of events and exploratory analyses and should not be regarded as confirmatory findings that supersede the FDR result for the composite indicator.

Finally, longitudinal changes in the microbiota before and after salpingectomy or proximal tubal occlusion and the association between GPAC detection and reproductive outcomes were not evaluated. Future prospective studies should combine quantitative assessment of bacterial burden, rigorous negative controls, paired vaginal, endometrial, and fallopian tube samples, histological assessment of inflammation, and reproductive outcome data.

## 5 Conclusion

In exploratory analyses, hydrosalpinx was associated with an increased frequency of detection of GPAC‐associated genera in the endometrial microbiota. In contrast, no clear associations were observed for the relative abundance of *Lactobacillus*, NLDM, NLPM, or the selected representative BV‐associated genera.

Although the association with GPAC detection did not meet the threshold for statistical significance after multiplicity adjustment, the direction of the effect estimates was maintained in sensitivity analyses using relative‐abundance thresholds of 0.5% and 1.0%, detection of at least two genera, and Firth regression. These findings suggest that alterations in the intrauterine environment associated with hydrosalpinx may manifest as a specific anaerobic bacterial signal that is not captured by broad measures of *Lactobacillus* predominance. These findings should be considered hypothesis‐generating rather than confirmatory and require independent prospective validation.

## Funding

This research received no specific grant from any funding agency in the public, commercial, or not‐for‐profit sectors.

## Ethics Statement

This study was approved by the Ethics Committee of Hanabusa Women's Clinic (Approval No. 2026–08) and was conducted in accordance with the Declaration of Helsinki and its later amendments.

## Consent

Comprehensive written informed consent for the potential secondary use of de‐identified clinical and test data for medical research and academic presentation was obtained from all patients. Because this was a retrospective observational study using existing clinical information and test results, the requirement for additional study‐specific informed consent was waived by the Ethics Committee of Hanabusa Women's Clinic.

## Conflicts of Interest

The authors declare no conflicts of interest.

## Supporting information


**Figure S1:** Sensitivity analyses of the association between hydrosalpinx and detection of GPAC‐associated genera using alternative operational definitions. Dots and horizontal lines indicate adjusted odds ratios and 95% confidence intervals from logistic regression models adjusted for age, antibiotic use within 3 months before testing, and luteal‐phase sampling. Counts are based on the full analysis cohort, whereas adjusted analyses included 229 complete cases. GPAC‐associated genera were operationally defined as *Anaerococcus*, *Finegoldia*, *Peptoniphilus*, *Peptostreptococcus*, and *Parvimonas*. Total GPAC relative abundance was calculated as the sum of the relative abundances of these five genera. Analyses using relative‐abundance thresholds and detection of at least two genera were conducted post hoc. *p* values were descriptive and were not adjusted for multiple comparisons.
**Table S1:** Sensitivity analyses of the association between hydrosalpinx and detection of GPAC‐associated genera across alternative outcome definitions and regression methods.
**Table S2:** Leave‐one‐genus‐out sensitivity analysis of the association between hydrosalpinx and detection of GPAC‐associated genera.
**Table S3:** Exploratory genus‐level associations between hydrosalpinx and detection of individual GPAC‐associated genera.

## Data Availability

The data that support the findings of this study are available on request from the corresponding author. The data are not publicly available due to privacy or ethical restrictions.
