# Supplementary material for: Association Between Hydrosalpinx and Detection of Gram‐Positive Anaerobic Cocci in the Endometrial Microbiota: A Single‐Center Retrospective Observational Study
Source: Reprod Med Biol. 2026 Oct 25;25(1):e70109. doi: 10.1002/rmb2.70109 (PMC13696902; doi:10.1002/rmb2.70109)
Supplement: Supplementary file 1 [file RMB2-25-e70109-s001.docx]

**Supporting Information**

**Association between Hydrosalpinx and Detection of Gram-Positive Anaerobic Cocci in the Endometrial Microbiota: A Single-Center Retrospective Observational Study**

Ryota Okamoto et al.

**Contents**
Supplementary Figure S1
Supplementary Table S1
Supplementary Table S2
Supplementary Table S3

**Supplementary Figure S1. Sensitivity analyses of the association between hydrosalpinx and detection of GPAC-associated genera using alternative operational definitions.** Dots and horizontal lines indicate adjusted odds ratios and 95% confidence intervals from logistic regression models adjusted for age, antibiotic use within 3 months before testing, and luteal-phase sampling. Counts are based on the full analysis cohort, whereas adjusted analyses included 229 complete cases. GPAC-associated genera were operationally defined as *Anaerococcus*, *Finegoldia*, *Peptoniphilus*, *Peptostreptococcus*, and *Parvimonas*. Total GPAC relative abundance was calculated as the sum of the relative abundances of these five genera. Analyses using relative-abundance thresholds and detection of at least two genera were conducted post hoc. *P* values were descriptive and were not adjusted for multiple comparisons.
**Abbreviations:** aOR, adjusted odds ratio; CI, confidence interval; GPAC, gram-positive anaerobic cocci.

Supplementary Table S1. Sensitivity analyses of the association between hydrosalpinx and detection of GPAC-associated genera across alternative outcome definitions and regression methods

| **GPAC outcome definition** | **Hydrosalpinx**  ***n*/*N* (%)** | **No hydrosalpinx *n*/*N* (%)** | **Conventional logistic regression** | | **Firth logistic regression** | |  |
| --- | --- | --- | --- | --- | --- | --- | --- |
|  |  |  | **aOR (95% CI)** | **P value** | **aOR (95% CI)** | **P value** | |
| Detection of any GPAC genus  (primary definition) | 23/90 (25.6) | 19/146 (13.0) | 2.20 (1.10–4.38) | 0.025 | 2.15 (1.09–4.25) | 0.027 | |
| Total GPAC relative abundance ≥0.5% | 12/90 (13.3) | 8/146 (5.5) | 2.75 (1.07–7.07) | 0.036 | 2.64 (1.07–6.51) | 0.036 | |
| Total GPAC relative abundance ≥1.0% | 9/90 (10.0) | 5/146 (3.4) | 3.41 (1.09–10.70) | 0.036 | 3.18 (1.10–9.23) | 0.033 | |
| Detection of ≥2 GPAC genera | 12/90 (13.3) | 6/146 (4.1) | 3.51 (1.26–9.78) | 0.016 | 3.30 (1.26–8.67) | 0.015 | |

Data are presented as *n*/*N* (%). Conventional and Firth bias-reduced logistic regression models were adjusted for age, antibiotic use within 3 months before testing, and luteal-phase sampling. Adjusted analyses included 229 complete cases. GPAC-associated genera were operationally defined as *Anaerococcus*, *Finegoldia*, *Peptoniphilus*, *Peptostreptococcus*, and *Parvimonas*. Total GPAC relative abundance was calculated as the sum of the relative abundances of these five genera. Analyses using relative-abundance thresholds and detection of ≥2 genera were conducted post hoc. *P* values were descriptive and were not adjusted for multiple comparisons.

Abbreviations: aOR, adjusted odds ratio; CI, confidence interval; GPAC, gram-positive anaerobic cocci.

Supplementary Table S2. Leave-one-genus-out sensitivity analysis of the association between hydrosalpinx and detection of GPAC-associated genera

| **Genus omitted from the GPAC definition** | **Hydrosalpinx**  ***n*/*N* (%)** | **No hydrosalpinx**  ***n*/*N* (%)** | **aOR (95% CI)** | **P value** |
| --- | --- | --- | --- | --- |
| None (primary definition) | 23/90 (25.6) | 19/146 (13.0) | 2.20 (1.10–4.38) | 0.025 |
| *Anaerococcus* | 19/90 (21.1) | 19/146 (13.0) | 1.69 (0.83–3.45) | 0.150 |
| *Finegoldia* | 19/90 (21.1) | 12/146 (8.2) | 2.68 (1.22–5.92) | 0.014 |
| *Peptoniphilus* | 20/90 (22.2) | 15/146 (10.3) | 2.60 (1.24–5.46) | 0.012 |
| *Peptostreptococcus* | 23/90 (25.6) | 18/146 (12.3) | 2.35 (1.17–4.74) | 0.017 |
| *Parvimonas* | 23/90 (25.6) | 18/146 (12.3) | 2.36 (1.17–4.75) | 0.016 |

None indicates the primary definition, in which GPAC positivity was defined as detection of at least one of the five GPAC-associated genera. In each leave-one-genus-out analysis, the indicated genus was omitted, and positivity was redefined as detection of at least one of the remaining four genera. Counts are based on the full analysis cohort. Logistic regression models were adjusted for age, antibiotic use within 3 months before testing, and luteal-phase sampling and included 229 complete cases. An aOR greater than 1 indicates higher odds of positivity in patients with hydrosalpinx. These analyses were conducted post hoc. *P* values were descriptive and were not adjusted for multiple comparisons.

Abbreviations: aOR, adjusted odds ratio; CI, confidence interval; GPAC, gram-positive anaerobic cocci.

Supplementary Table S3. Exploratory genus-level associations between hydrosalpinx and detection of individual GPAC-associated genera

| **GPAC-associated genus** | **Hydrosalpinx**  ***n*/*N* (%)** | **No hydrosalpinx**  ***n*/*N* (%)** | **Firth logistic regression** | | |  |
| --- | --- | --- | --- | --- | --- | --- |
|  |  |  | **aOR (95% CI)** | **P value** | **q value** | |
| *Anaerococcus* | 10/90 (11.1) | 3/146 (2.1) | 5.54 (1.66–18.43) | 0.005 | 0.026 | |
| *Finegoldia* | 16/90 (17.8) | 12/146 (8.2) | 2.53 (1.14–5.61) | 0.023 | 0.038 | |
| *Peptoniphilus* | 15/90 (16.7) | 8/146 (5.5) | 2.91 (1.21–7.04) | 0.018 | 0.038 | |
| *Peptostreptococcus* | 5/90 (5.6) | 2/146 (1.4) | 3.58 (0.87–14.74) | 0.078 | 0.097 | |
| *Parvimonas* | 0/90 (0.0) | 2/146 (1.4) | 0.33 (0.03–4.15) | 0.390 | 0.390 | |

Data are presented as *n*/*N* (%). Firth bias-reduced logistic regression models were adjusted for age, antibiotic use within 3 months before testing, and luteal-phase sampling and included 229 complete cases. The five genus-level analyses were treated as a separate exploratory multiplicity family. *P* values were adjusted using the Benjamini–Hochberg procedure to control the false discovery rate across the five genera, and the resulting *q* values are presented. An aOR greater than 1 indicates higher odds of detection in patients with hydrosalpinx.

Abbreviations: aOR, adjusted odds ratio; CI, confidence interval; GPAC, gram-positive anaerobic cocci.
